# Low sequence diversity of the prion protein gene (*PRNP*) in wild deer and goat species from Spain

**DOI:** 10.1186/s13567-018-0528-8

**Published:** 2018-04-10

**Authors:** José Luis Pitarch, Helen Caroline Raksa, María Cruz Arnal, Miguel Revilla, David Martínez, Daniel Fernández de Luco, Juan José Badiola, Wilfred Goldmann, Cristina Acín

**Affiliations:** 1 Centro de Encefalopatías y Enfermedades Transmisibles Emergentes, Facultad de Veterinaria, Universidad de Zaragoza, Zaragoza, Spain; 2 Departamento de Patología Animal, Facultad de Veterinaria, Universidad de Zaragoza, Zaragoza, Spain; 3 The Roslin Institute and Royal (Dick) School of Veterinary Studies, University of Edinburgh, Easter Bush, Midlothian, UK

## Abstract

The first European cases of chronic wasting disease (CWD) in free-ranging reindeer and wild elk were confirmed in Norway in 2016 highlighting the urgent need to understand transmissible spongiform encephalopathies (TSEs) in the context of European deer species and the many individual populations throughout the European continent. The genetics of the prion protein gene (*PRNP*) are crucial in determining the relative susceptibility to TSEs. To establish *PRNP* gene sequence diversity for free-ranging ruminants in the Northeast of Spain, the open reading frame was sequenced in over 350 samples from five species: Iberian red deer (*Cervus elaphus hispanicus*), roe deer (*Capreolus capreolus*), fallow deer (*Dama dama*), Iberian wild goat (*Capra pyrenaica hispanica*) and Pyrenean chamois (*Rupicapra p. pyrenaica*). Three single nucleotide polymorphisms (SNPs) were found in red deer: a silent mutation at codon 136, and amino acid changes T98A and Q226E. Pyrenean chamois revealed a silent SNP at codon 38 and an allele with a single octapeptide-repeat deletion. No polymorphisms were found in roe deer, fallow deer and Iberian wild goat. This apparently low variability of the *PRNP* coding region sequences of four major species in Spain resembles previous findings for wild mammals, but implies that larger surveys will be necessary to find novel, low frequency *PRNP* gene alleles that may be utilized in CWD risk control.

## Introduction

Transmissible spongiform encephalopathies (TSEs) are a group of fatal, neurodegenerative disorders characterised by the accumulation in the central nervous system of prion protein PrP^Sc^, an abnormal isoform of the cellular protein PrP^C^ [1, 2]. TSEs can affect several mammalian species, but show a predominance in ruminants: scrapie in sheep and goats, bovine spongiform encephalopathy (BSE) in bovids and chronic wasting disease (CWD) in cervids.

Scrapie is a widespread disease known for more than 250 years, which is present in almost all regions of the world [3], while BSE reached epidemic proportions in Europe in the 1990s due to the use of animal feedstuffs contaminated with prions [4]. Both TSEs affected livestock and exotic ruminants in zoological collections, but there is no evidence that any wild and free-ranging ruminants suffered natural scrapie or BSE.

In contrast, CWD has affected North American free-ranging mule deer, white tailed deer and wapiti. Originally centred in Colorado, Wyoming and Southwest Canada [5–7], CWD has now spread through many US states, mostly in captive wapiti and deer, including a captive red deer from a herd located in Minnesota [8]. Additionally, chronic wasting disease has been orally transmitted to Shira’s moose [9], red deer [10], reindeer [11] and Reeves’ muntjac deer [12], but only by intracerebral inoculation to fallow deer [13]. Although natural transmission of CWD to humans seems unlikely, several studies recommend establishing preventive measures and further research on the subject [14, 15].

The presence of CWD in Europe was confirmed recently: reindeer (*Rangifer tarandus*) and European elk (*Alces alces*) were diagnosed in two separated regions of Norway [16, 17]. There is no evidence for CWD among other European deer populations [18].

Susceptibility to any TSE depends largely on the PrP^C^ sequence, encoded by the host *PRNP* gene. Several studies of natural and experimental scrapie infection in sheep and goats showed how allelic variations in the *PRNP* gene modulates disease susceptibility [19, 20], and it is therefore critical to consider the *PRNP* gene sequence in wild ruminants for the assessment of TSE infection risk.

Studies of CWD in North America have indicated several amino acid substitutions in PrP^C^ that are associated with different susceptibility to disease. In wapiti (or Rocky Mountain elk, *Cervus canadensis nelsoni*) polymorphism M132L appears to modulate disease [21–24], in mule deer (*Odocoileus hemionus*) it is polymorphism S225F [25, 26] and in white-tailed deer (*Odocoileus virginianus*) polymorphisms G96S, Q95H and A116G are modulators [27–29]. Only limited genotype survey data exist for European deer populations. Similarly, a survey between the years 2007 and 2009 of 537 wild red deer from Spain tested negative for the presence of PrP^Sc^ as did all other European surveys conducted in parallel. Since then, no further diagnostic reports have been published for any cervid in Spain.

The area of this study was located at the Northeast of Spain, between the mountain chain of the Pyrenees and the Iberian System, spanning the whole region of Aragon, part of La Rioja and the eastern side of Castilla y Leon. In these locations, there are five mayor species of wild ruminants: Iberian red deer (*Cervus elaphus hispanicus*), roe deer (*Capreolus capreolus*), fallow deer (*Dama dama*), Iberian wild goat (*Capra pyrenaica hispanica*) and Pyrenean chamois (*Rupicapra p. pyrenaica*). Pyrenean chamois and Iberian wild goat belong to the family *Bovidae*, subfamily *Caprinae* and are theoretically at risk to scrapie or BSE infection. Red deer, fallow deer and roe deer represent two subfamilies of cervids (*Cervinae* and *Capreolinae*) which are at risk CWD infection.

An estimation of the total population of the different species in the North East area has been revised. In this sense, the population estimated for the deer population is about 6500 individuals. More prolific and represented is the roe deer with 23 600 individuals and finally totally of 13 000 and 7200 is the estimation for Pyrinean Chamois and Iberian wild goat [30, 31].

The aim of this work was to study the *PRNP* gene of the different species of wild ruminants of the Northeast area of Spain in order to evaluate their status of resistance or susceptibility to prion diseases.

## Materials and methods

### Animals and samples

**Figure 1:**
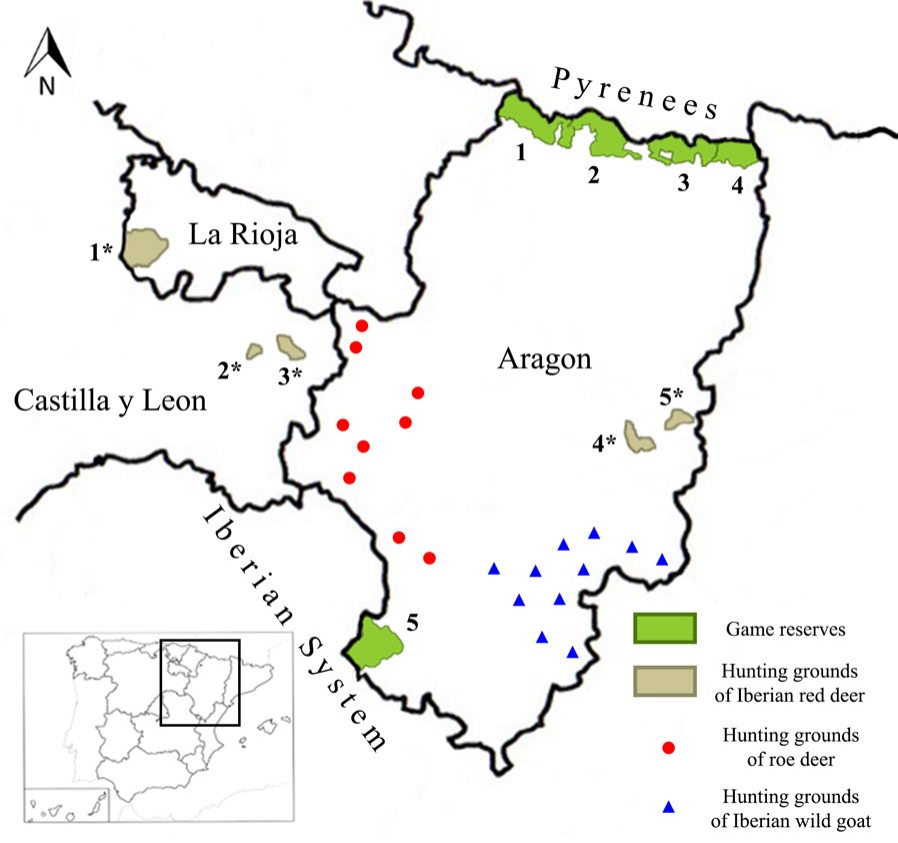
**Map of the northeast of Spain showing the geographic locations where different samples were obtained.** Game reserves: 1, Los Valles; 2, Viñamala; 3, Los Circos; 4, Benasque; 5, Montes Universales. Hunting grounds of Iberian red deer: 1*, Ezcaray; 2*, Renieblas; 3*, Arancón; 4*, Caspe; 5*, Fraga. Reed deer: Arancon (*n* = 13), Renieblas (*n* = 9) Ezcaray (*n* = 48) Caspe (*n* = 50) Fraga (*n* = 78) and Montes Universales (*n* = 11). Fallow deer: Montes Universales (*n* = 15). Roe deer: Los Valles (*n* = 2), Viñamala (*n* = 10), Los Circos (*n* = 4), Benasque (*n* = 15) and West Aragon (*n* = 13). Pyrenean chamois: Los Valles (*n* = 11), Viñamala (*n* = 9), Los Circos (*n* = 13) and Benasque (*n* = 20).

This study includes 351 animals of five different species of wild ruminants: Iberian red deer (*Cervus elaphus hispanicus*), roe deer (*Capreolus capreolus*), fallow deer (*Dama dama*), Iberian wild goat (*Capra pyrenaica hispanica*) and Pyrenean chamois (*Rupicapra p. pyrenaica*). All of them were free-ranging animals from different places of northeast of Spain, and were hunted or found dead in game reserves or hunting grounds (Figure 1).

Red deer (*n* = 209) were grouped into three areas according to their geographical proximity. The west area (WA) combined 70 animals from the hunting grounds of Arancon (*n* = 13), Renieblas (*n* = 9) and Ezcaray (*n* = 48). The east area (EA) combined 128 animals from the hunting grounds of Caspe (*n* = 50) and Fraga (*n* = 78). The south area (SA) is represented by 11 animals from the game reserve of Montes Universales; this area was also the origin of the fallow deer samples (*n* = 15). Roe deer and Pyrenean chamois (*n* = 44; *n* = 53) came from the Pyrenean game reserves of Los Valles (*n* = 2; *n* = 11), Viñamala (n = 10; *n* = 9), Los Circos (*n* = 4; *n* = 13) and Benasque (*n* = 15; *n* = 20); and from several hunting grounds of West Aragon (*n* = 13). Iberian wild goats came from different hunting grounds located in South-East Aragon (*n* = 30). Spleen samples from all animals of the study were collected and kept frozen (−20 °C) in order to extract the genomic DNA. Samples of obex, tonsil or ileocecal valve were collected from 68 red deer from the hunting grounds of Caspe (*n* = 32), Fraga (*n* = 26) and Renieblas (*n* = 10), they were preserved in 10% neutral buffered formaldehyde and embedded in paraffin for TSE diagnosis.

### Extraction and purification of genomic DNA

Genomic DNA was extracted from 500 mg of spleen using a QIAamp DNA mini kit (QIAGEN^®^) following the manufacturers protocol. In brief, two digestions of 1 h at 56 °C with proteinase K (20 ng/mL) lysed the cells of the tissue. Then, by using a membrane column, ethanol was added to assist the precipitation of DNA and binding to the membrane. Finally, a series of washes removed debris and then purified genomic DNA was recovered.

### *PRNP* gene amplification and sequencing

The open reading frame (ORF) of *PRNP* gene (771 bp) of all animals except fallow deer was amplified by performing a PCR using the reagents of the commercial kit of QIAGEN^®^ (HotStarTaq^®^ Master Mix Kit) and primers SILV-8 (fwd) (5′-AAAGCCACATAGGCAGCTGGAT-3′) and SILV-778 (rev) (5′-AGAAGATAATGAAAACAGGAAG-3′) [21] for roe and red deer; and PrP8 (fwd) (5′-CAGGTTAACGATGGTGAAAAGCCACATAGG-3′) and PrP9 (rev) (5′-GGAATTCTATCCTACTATGAGAAAAATGAGG-3′) [32] for Iberian wild goat and Pyrenean chamois. PCR reactions were purified using the vacuum manifold from Millipore^®^. Bi-directional sequencing was performed using the same PCR primers. Chromatograms were analysed using BioEdit v.4.8.6.

Fallow deer samples were PCR amplified with AmpliTaq Gold360 (Thermo Fisher Scientific) using either primer −143d (ATGGAATGTGAAGAACATTTATGACCTA) or primer −213d (AGGTCAACTTTGTCCTTGGAGGAG) in combination with primer +139u (TAAGCGCCAAGGGTATTAGCAT). Sequences were generated as described in Goldmann et al. [33] with oligonucleotide +70u GCTGCAGGTAGATACTCCCTC.

New sequences were deposited in Genbank with the following accession numbers: *Cervus elaphus hispanicus* KT845862-KT845864, *Capra pyrenaica hispanica* KT845865, *Rupicapra pyrenaica pirenaica* KT845866-KT845868.

### Statistical analysis

The results obtained for different subpopulations are compared statistically using the Fisher exact test of 2 × 2 and 2 × 3 contingency tables, with *p* < 0.05 considered significant. The computer program GENEPOP was used to perform a statistical test to determine possible deviations from the Hardy–Weinberg equilibrium.

### PrP^sc^ immunohistochemical detection and haematoxylin-eosine stain

Sections of 5 µm of formaldehyde-fixed and paraffin-wax-embedded lymphoid tissues and obex were subjected to immunohistochemical diagnosis of CWD using MAb F99/97.6.1, a mouse monoclonal antibody antiPrP [34]. In addition, tissue sections were stained with haematoxylin and eosin (HE), in order to observe possible histopathological lesions.

## Results

### Deer *PRNP*

The coding region of the *PRNP* gene from 209 Iberian red deer samples collected in North-East Spain showed three single-nucleotide polymorphisms (SNPs): a silent SNP at position 408 (codon 136) (gct→gcc), and two polymorphisms at positions 292 (acc→gcc) and 776 (cag→gag), resulting in amino acid changes in codons 98 and 226. The mutation in codon 98 causes an amino acid substitution of threonine (T) with alanine (A), while in codon 226 the mutation results in an amino acid change from glutamine (Q) to glutamic acid (E). The SNP at nucleotide position 408 is linked to the SNP at position 776, so that all haplotypes were either t408-a776(Q_226_) or c408-g776(E_226_); Tables 1 and 2 only show the amino acid changes. A_98_ was observed 61 times in linkage with Q_226_, and all AA_98_ homozygous genotypes were QQ_226_ homozygous. Therefore, it is assumed that A_98_ was also linked with Q_226_ in the 28 TA_98_-QE_226_ heterozygotes so that their genotype can be described as TE/AQ. The three haplotypes described here for Spanish red deer were identical to haplotypes 1, 8 and 10 found in Italy and Scotland [35].

**Table 1:** **Genotypic frequencies (%) of** ***PRNP*** **polymorphisms in Iberian red deer from the North-East of Spain**

Codon 98	Codon 226	Genotypes	Number	Frequencies (%)			
Thr(T) Ala(A)	Gln(Q) Glu(E)			Total	EA	WA	SA
TT	QQ	TQ/TQ	44	21.05	18.8	24.3	27.3
TT	QE	TQ/TE	56	26.8	23.4	30	45.4
TT	EE	TE/TE	33	15.8	14.1	17.1	27.3
TA	QQ	TQ/AQ	35	16.75	19.5	14.3	0
AA	QQ	AQ/AQ	13	6.2	7.8	4.3	0
TA	QE	TE/AQ	28	13.4	16.4	10	0
–	–	TOTAL	209	100	100	100	100

T: threonine, A: alanine, Q: glutamine, E: glutamic acid, EA: east area, WA: west area, SA: south area.

**Table 2:** **Allelic frequencies (%) of** ***PRNP*** **polymorphisms in EA and WA subpopulations of Iberian red deer**

Alleles Codons 98-226	Haplotype in Peletto et al. [34]	Number	Allele Frequencies (%)			
			Total	EA	WA	SA
TQ	1	179	42.8	40.3	46.4	50
TE	10	150	35.9	34	37.2	50
AQ	8	89	21.3	25.7	16.4	0
Total		418	100	100	100	100

T: threonine, A: alanine, Q: glutamine, E: glutamic acid, EA: east area; WA: west area, SA: south area.

The SNPs in codons 136 and 226 appeared in all the three geographic areas, while the polymorphism at codon 98 appeared in deer from EA and WA, but not SA. The allele and genotype frequencies of the amino acid polymorphisms were calculated for the whole population, and separately for the different subpopulations in EA, WA and SA (Tables 1 and 2). Allele frequencies for the TQ and TE alleles were 74.3% in the EA, 83.6% in the WA and 100% in the SA. Correspondingly, frequencies for the AQ allele were 25.7% in the EA, 16.4% in the WA and 0% in the SA. The lack of the AQ allele from the SA population is significant compared with the EA (*p* = 0.003) and WA (*p* = 0.045) populations although only 11 animals were collected. When the EA and WA populations are compared for the AQ allele frequencies, the difference of 9.3% was also significant (*p* = 0.03). However, there was no significant difference between TQ and TE alleles when any of the three populations were compared; on average both alleles appear balanced at 53.2% and 46.8%, respectively. Both subpopulations EA and WA are in HWE for the three SNPs. When the allele frequencies of the Alpine red deer *Cervus elaphus elaphus* (AQ:TQ:TE = 10%:62%:28%) [35] was compared to the Spanish red deer *Cervus elaphus hispanicus* (21.3%:42.8%:35.9%) the differences were significant (*p* = 0.03), with an increased frequency of the AQ allele.

The 15 fallow deer *PRNP* coding region sequences (codon 23-256) showed no SNP at all, matching the red deer haplotype t408-a292(T_98_)-g776(E_226_), but with the previously described codon 138 sequence aat (N_138_) deposited in Genbank (accession no. AY286007). Similarly, all 44 roe deer *PRNP* coding region sequences were identical to each other and to the published sequence of roe deer (Accession No. AY639096). The roe deer allele is identical to the red deer TQ allele.

### Iberian wild goat and chamois *PRNP*

The *PRNP* allele deduced from 30 Iberian wild goat sequences was identical to the Alpine ibex (*Capra ibex ibex*, accession no. EF139174) and Nubian ibex (*Capra ibex nubiana*, AF117319). It showed no polymorphisms. Finally, the *PRNP* coding region sequences of 53 Pyrenean chamois revealed the presence of a silent mutation at position 114 (gga→ggg, codon 38) and a 24 bp deletion, but was otherwise identical to Alpine chamois (Accession No. AY735496.1). The g114 haplotype was found at a frequency of 13.2%. The 24 bp deletion (tcagccccatggaggtggctgggg) causes the loss of one octapeptide (PHGGGWGQ) of the standard five repeats in the N-terminal region of the wild type PrP protein, it was observed in eight heterozygous animals (allele frequency 0.9%).

### Histological analysis for PrP^sc^ deposition in red deer

**Figure 2:**
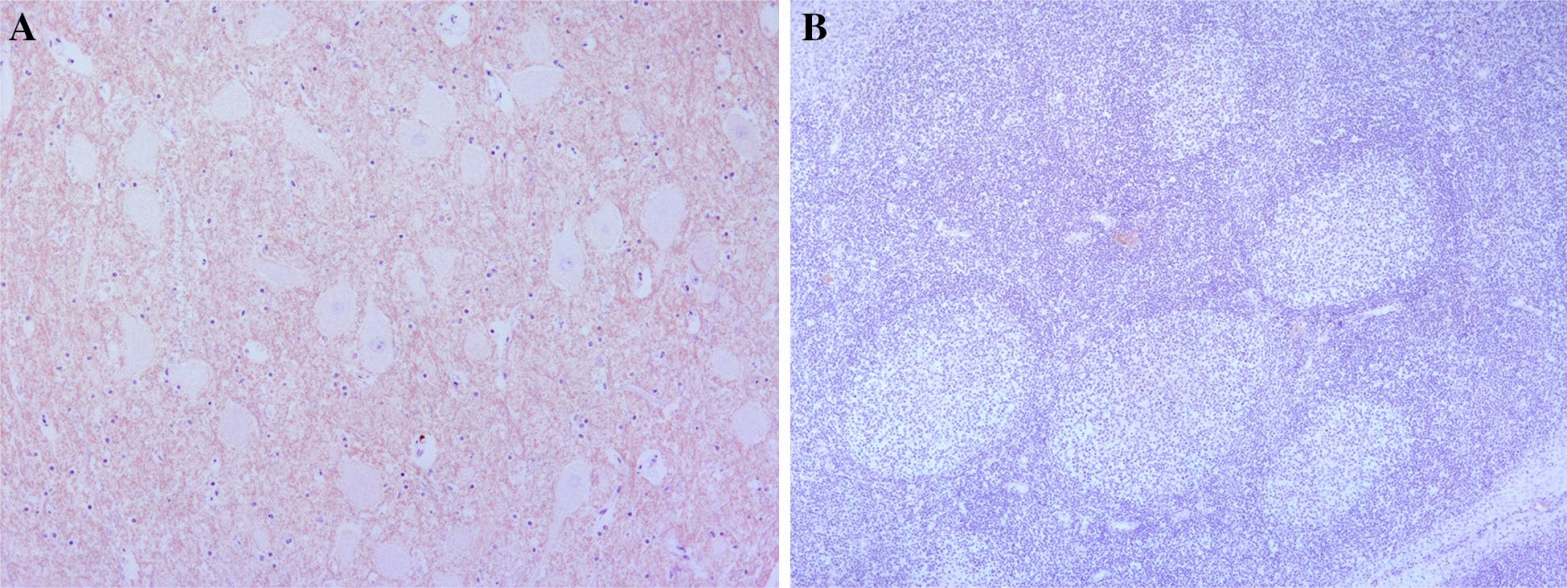
**Absence of PrP**^**sc**^ **in central nervous system and lymphoreticular system of Iberian red deer.** Absence of deposition of PrP^sc^ in histological sections of brain at level of dorsal motor nucleus of the vague nerve (10×) (**A**), and tonsil (10×) (**B**) of an Iberian red deer by immunohistochemistry using antibody F99/97.6.1.

PrP^Sc^ immunoreactivity was assayed in obex and lymphoid tissue (tonsil and/or ileocecal valve) in 54 animals and in lymphoid tissue only in an additional 14 animals. Animals of all genotypes were selected: TE/AQ (14), AQ/TQ (18), TE/TE (8), TQ/TQ (12) and TE/TQ (16). None of these 68 animals showed any PrP^Sc^ in any of the tissues (Figure 2). Other, non-TSE histological observations included CNS injuries that may be associated with the time of death of the animal, such as perivascular haemorrhages, observed in 35% of the animals. Furthermore, the presence of mild lymphoplasmacytic perivascular cuffing and gliosis foci was detected in 15%, not associated to any particular cause such as unspecific inflammation. Anyway, no microscopic lesion that could correspond to those characteristics of TSE was observed in the brain of the animals.

## Discussion

The analysis of 700 *PRNP* gene coding regions from three cervine and two wild caprine species revealed only three amino acid polymorphisms in PrP^C^, and an additional two synonymous SNPs. Whilst the cervine polymorphisms have been described before, the two polymorphisms in the chamois were new.

The two amino acid substitutions T98A and Q226E detected in Iberian red deer have both previously been described in an Italian red deer *PRNP* gene survey, in the same allele combination of AQ, TQ and TE. There is a significant difference in the allele frequencies between the Italian, British and Spanish populations but its relevance for CWD remains to be investigated [35]. The *PRNP* polymorphism M132L described in the related North American wapiti (Rocky mountain elk) has not been found yet in any European red deer [21, 24].

Threonine in codon 98 appears to be the most commonly found amino acid in this position, including human PrP^C^. While a serine substitution in codon 98 is found in sheep, goats and muntjac deer amongst many others, the replacement with alanine is much rarer, seen only in red deer, camel, two monkey species and armadillo. Although the T-S-A substitutions have to be regarded as conservative, they may still have a role in the conversion of PrP^C^ into the pathogenic form PrP^Sc^ that is found in CWD and all other prion diseases. This codon position is not only very close to the proteinase K cleavage site of the partially resistant PrP^Sc^ protein fragment, it is also near codons 95 and 96, which are associated with susceptibility to CWD of white-tailed and mule deer [27–29].

Codon 226 is located in the third α-helix of PrP^C^ and remains part of the proteinase K resistant fragment of PrP^Sc^. It may therefore play a role in the stability and conversion of PrP^C^. Indeed, many amino acid substitutions in helix 3, particularly in human PrP^C^, have been shown to change the propensity for prion disease development. On the other side, substitutions in codon 222 in caprine PrP or the equivalent position 219 in human PrP are associated with strong disease resistance. Equally, polymorphism S225F seems to play a role in CWD resistance in mule deer [25]. In white-tailed deer another codon 226 polymorphism, Q226K, was found at low frequency [29].

Recent experimental CWD challenge studies in red deer have been performed which coincidentally used animals with all three codon 226 genotypes (QQ_226_, QE_226_, EE_226_). There were no significant differences in PrP^Sc^ western blot profiles or in the incubation times associated with those genotypes, but the number of animals in the study was far too small to come to a significant conclusion regarding genetic association [10]. Another experiment showed that European red deer with QQ226 genotype were susceptible to intracerebral challenge with BSE brain homogenate [36]. A similar study using BSE by oral route let to disease in a QQ_226_ deer, while the other genotypes (EE_226_, QE_226_) remained healthy [37]. Then again, the number of animals with particular genotypes was too small to establish genetic association with susceptibility or pathogenesis.

The analysis for disease-associated deposition of PrP^Sc^ by immunocytochemistry in cervine tissues from 68 animals has given a negative result, which at least indicates that CWD is not hiding as non-clinical disease epidemic in Spanish red deer. Of course many more samples need to be studied to allow for a definite assessment regarding the absence of CWD from these and neighbouring populations.

No *PRNP* sequence variation was found in approx. 120 chromosomes analysed for Iberian roe and fallow deer, which is not dissimilar to the Italian and Swedish studies, which revealed one synonymous polymorphism in approx. 420 chromosomes between them [35, 38]. Only further extensive genotyping of these species will prove whether they have indeed a particularly low *PRNP* coding region variability.

Animals from the subfamily *Caprinae*, like Pyrenean chamois and Iberian wild goat, are thought to be susceptible to scrapie because of the close resemblance of their *PRNP* sequence with domestic goat *PRNP* [39]. The deletion in the octapeptide region observed in one Pyrenean chamois is a novel polymorphism for this species, but octapeptide repeat deletions in PrP^C^ have been found regularly in several species. Approximately 10% of PrP^C^ from over 140 species have shown deletions of one, two or even three octapeptides, whilst the opposite, a single octapeptide insertion has been found in 15% of PrP alleles. Whether an association exists between octapeptide number variation and TSE in ruminants remains unresolved.

CWD, the most important TSE affecting cervids, has been confined to North America for years, but finally it has reached the European continent. Its great capacity for dissemination means that, eventually it could reach other regions from Europe, including the Iberian Peninsula. Besides, a real contact with the causal agent of scrapie occurs constantly because of the extensive grazing areas shared by the population of wild ruminant, especially red deer, and sheep and goats flocks. In addition, the great capacity of prion to jump between species [40] poses a risk to these deer [41, 42].

This survey has shown that the *PRNP* gene is highly conserved in four important species of wild ruminants in the northeast of Spain and supports the current assumption that there is no hidden TSE epidemic in red deer.

## Abbreviations

CWD: chronic wasting disease
TSE: transmissible spongiform encephalopathy
PRNP: prion protein gene
SNP: single nucleotide polymorphism
BSE: bovine spongiform encephalopathy
WA: west area
EA: east area
SA: south area
